# Conventional biventricular pacing is still preferred to conduction system pacing for atrioventricular block in patients with reduced ejection fraction and narrow QRS

**DOI:** 10.1093/europace/euad337

**Published:** 2023-12-28

**Authors:** Michael Glikson, Marek Jastrzebski, Michael R Gold, Kenneth Ellenbogen, Haran Burri

**Affiliations:** Jesselson Integrated Heart Center, Shaare Zedek Medical Center and Hebrew University Faculty of Medicine, Jerusalem, Israel; First Department of Cardiology, Interventional Electrocardiology and Hypertension, Jagiellonian University, Medical College, Jakubowskiego 2, 30-688 Krakow, Poland; Virginia Commonwealth University, VCU Medical Center Gateway Building, 1200 E. Marshall Street, Richmond, VA 23219, USA; MUSC Division of Cardiology, Medical University of South Carolina, 25 Courtenay Dr, MS-592, Charleston, SC 29425, USA; Cardiac Pacing Unit, Cardiology Department, University Hospital of Geneva, Rue Gabrielle Perret Gentil 4, 1211, Geneva, Switzerland

**Keywords:** Cardiac pacing, Biventricular pacing, Atrioventricular block, Narrow QRS, Heart failure, Conduction system pacing, His bundle branch pacing, Left bundle branch area pacing, Cardiac resynchronization therapy

## Abstract

It is well established that right ventricular pacing is detrimental in patients with reduced cardiac function who require ventricular pacing (VP), and alternatives nowadays are comprised of biventricular pacing (BiVP) and conduction system pacing (CSP). The latter modality is of particular interest in patients with a narrow baseline QRS as it completely avoids, or minimizes, ventricular desynchronization associated with VP. In this article, experts debate whether BiVP or CSP should be used to treat these patients.

## Graphical abstract

**Graphical abstract:**
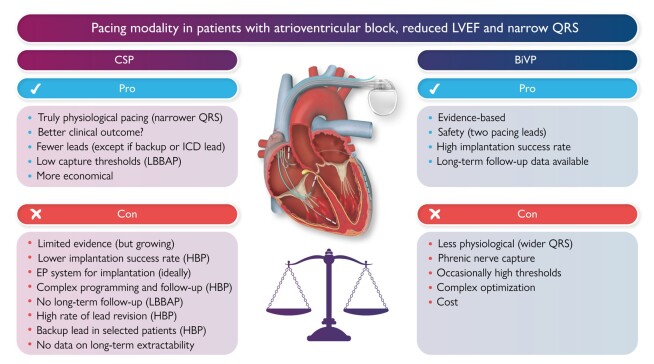

## Introduction

### Haran Burri

It is a well-established fact that right ventricular pacing (RVP) may result in pacing-induced cardiomyopathy (PICM) with decreased left ventricular ejection fraction (LVEF), occurring after 5 years in roughly 15–20% of patients with normal baseline ventricular function who have >20% pacing.^1,2^ Clinical outcome in patients with baseline reduced LVEF is worsened by RV apical pacing.^3^ In the quest for providing more physiological stimulation, RV pacing of the interventricular septum has been evaluated but has not been shown to be superior to apical pacing in randomized studies^4,5^ and does not appear to confer any clinical benefit beyond avoidance of perforation of the free wall.^6,7^ Biventricular pacing (BiVP) has however been shown by randomized trials to be superior to RV pacing in patients with reduced LVEF who required antibradycardia pacing for atrioventricular block (AVB), with improved quality of life, NYHA class, and echocardiographic response.^8–10^ However, the baseline QRS in all these trial was on average >120 ms and part of the beneficial effect may be explained by the effect of *resynchronization* by BiVP in these patients. Conduction system pacing (CSP) is gaining mainstream practise^11–13^ and avoids *desynchronizing* ventricular activation in patients with a narrow baseline QRS. Currently, the European Society of Cardiology (ESC) guidelines on pacing recommend BiVP rather than RV pacing (Class I, level of evidence A) for patients with LVEF <40% who require antibradycardia ventricular pacing, regardless of QRS width, and heart failure (HF) symptoms. There was no indication for CSP in this patient population due to limited data at the time of writing of the document. The Heart Rhythm Society (HRS) has recently published guidelines on physiological pacing to avoid heart failure and have included His bundle pacing (HBP) and left bundle branch area pacing (LBBAP) with the same level of recommendation as BiVP for antibradycardia pacing in patients with LVEF 36–50%, irrespective of baseline QRS width (2a, level of evidence B).^14^ There is no guidance in this document for the type of antibradycardia pacing which is recommended in patients with LVEF <35%.

Both BiVP and CSP require proper implantation technique and adequate training. Coronary sinus leads are usually targeted in postero-lateral tributaries and in a non-apical position with adequate thresholds, absence of phrenic nerve capture and a stable position; it can sometimes be challenging to obtain all these criteria. Conduction system pacing also has its challenges. With HBP, Locating the His bundle and fixating the lead in a stable position with acceptable thresholds can be difficult. With LBBAP, penetrating the interventricular septum, recognizing microperforation and confirming conduction system capture (which is probably important in heart failure patients) can also be difficult. The EHRA consensus document on CSP implantation^13,15^ provides a framework for standardizing the procedure, but proper training (e.g. peer-to-peer or simulator-based) are probably also necessary to gain sufficient expertise to deliver safe and effective therapy.

Whereas we currently have randomized studies comparing BiVP and CSP for treating heart failure with bundle branch block,^16–20^ there are no studies comparing these treatment modalities in patients with AVB (other than in the setting of atrioventricular nodal ablation^21^). Patients with a narrow QRS and reduced LVEF are a particular therapeutic conundrum, for which pros and contras of CSP vs. BiVP are summarized in the graphical abstract. These points are further discussed and debated below.

## Pro

### Michael Glikson, Michael R Gold

#### Biventricular pacing to prevent pacing-induced cardiomyopathy

Treatment of AVB by BiVP in patients with reduced LVEF has been evaluated in three randomized trials,^8–10^ as well as several other observational studies that compared this pacing modality to RV apical pacing. In the most important seminal trial of this patient group (Block HF), Curtis *et al.*^9^ enrolled 691 patients with AVB with an LVEF ≤ 50%. All patients received a BiVP device and were randomized to RV apical pacing vs. BiVP. The primary composite outcome was time to death from any cause, an urgent care visit for HF that required intravenous therapy or an increase of 15% in left ventricular end-systolic volume (LVESV) index. The primary outcome was reached in 55.6% of the RV pacing compared to 45.8% in the BiV group (hazard ratio 0.74). Kindermann *et al.*^8^ in the HOBIPACE trial performed a cross-over study of RV pacing vs. BiV pacing in 30 patients with indication for pacing and LV below 40% of LV end-diastolic diameter > 60 mm. Biventricular pacing was superior in terms of LV function, quality of life, and exercise capacity. Martinelli *et al.*^10^ in the COMBAT trial randomized 60 patients with symptomatic HF, LVEF < 40%, and AVB to BiVP vs. RV apical pacing using a 3 months cross-over design. Quality of life, NYHA class LVEF, and LVESV were all better with BiVP. Notably, all three trials enrolled patients with a wide range of QRS width (average QRS width was 120, 170, and 150 ms in Block HF, Hobipace, and Combat, respectively). Details are not available on whether the basal QRSs were measured in sinus or in AV block and no separate analyses were provided for wide and for narrow QRS cases in any these trials.

Data of BiVP in patients with normal or preserved LVEF are less convincing in terms of benefit, with conflicting results and no apparent effect on survival, but with an effect on reverse remodelling.^22–25^ Yu *et al.*^24^ randomized, in the PACE trial, 177 patients with a normal LVEF at baseline who were implanted with biventricular pacemakers, to receive BiVP or RV apical pacing. After 1 year, LVEF was significantly lower in the RV apical pacing group (54.8% vs. 62.2%, *P* < 0.001) and the LVESV was higher (35.7 mL vs. 27.6 mL, *P* < 0.001), without differences in clinical outcome. However, over a follow-up of up to 5 years there was a significantly better LVEF in the BiVP group and a lower rate of HF hospitalizations.^25^ Albertsen *et al.*^26^ randomized 50 patients with normal baseline LVEF into RV pacing vs. BivP. Whereas there was a significant decrease in LVEF over 3 years in the RV group there was mild increase in the BiVP group. Doshi *et al.*^27^ in the PAVE study randomized 184 patients undergoing atrioventricular nodal ablation for intractable atrial fibrillation (AF) to RV pacing vs. BiVP. At 6 months, BiVP patients had better 6-minute walk test and better LVEF. Brignole *et al.*^28^ randomized 186 patients undergoing AV nodal ablation who had cardiac resynchronization therapy (CRT) devices to receive BiVP vs. RV apical pacing. During a median follow-up of 20 months, a composite endpoint of death or hospitalization or worsening due to HF occurred in 11% of the BiVP group compared to 26% of the RV apical pacing.

*Table 1* lists the main studies comparing BiVP to RV pacing in AV block. We can therefore conclude that there is a strong amount of information obtained in well-designed trials to support BiVP as a mean to prevent PICM in patients with all ranges of QRS and reduced LVEF who need pacing for AVB. Based on this evidence, the 2021 ESC pacing guidelines recommended BiVP rather than RV pacing in all patients with LVEF <40% who have an indication for pacing with AVB in order to reduce morbidity. This applied to both patients in sinus rhythm and in AF.^30^

**Table 1:** Studies of biventricular CRT vs. RV apical pacing for AV block

Author/acronym/reference	Design	Inclusion criteria	*n* with CSP	Follow-up duration	Findings BiVP compared to RVP
Curtis Block HF^9,29^	RCT of RVP vs. BiVP (software randomization)	AVB pacing indication LVEF ≤ 50%	349	37 mo	45.8% vs. 55.6% of death, HF or LV remodelling
Kinderman	Cross-over between BiVP and RVP	Standard pacing indication LVEF < 40% or LVEDD > 60	30	3 mo each arm	Reduced LV volumes, NTpro BNP, MLHF and increased EF and peak VO2
Hobipace^8^					
Martinelli	Cross-over between RVP and BiVP then left at one mode	AVB with LVEF < 40% NYHA II—IV	60	3 mo each arm, 17.5 mo	Significant improvement of LVEF, QOL, NYHA, LVESV
COMBAT^10^					
Yu	Randomized comparison of RVP and BiVP	Pacing indication with normal LVEF	89	12 mo/4.8 yr	Better LVEF, LVESV
PACE^24,25^					
					Lower HFH
Stockburger	Randomized comparison of RVP and BiVP	Patients with expected VP > 80%	50	12 mo	No difference in LV volumes
Prevent HF^22^					
Funck	Randomized comparison of RVP and BiVP	Patients with AVB, all LVEFs	902	5.6 yr	No difference in mortality/HFH ^a^
BioPace^23^					
Albertsen^26^	Randomized comparison of RVP and BiVP	High grade AVB, normal LVEF	25	3 yr	Better LVEF but no difference in clinical HF
Doshi PAVE^27^	Randomized comparison of RVP and BiVP	Patients undergoing AVN ablation all LVEF	103	6 mo	Better 6 min walk, LVEF; especially in patients with baseline LVEF < 45% of NYHA II/III
Brignole^28^	Randomized comparison of RVP and BiVP	Patients undergoing AVN ablation all LVEF s	97	20 mo	Better combined endpoint (death or hospitalization or worsening of HF

^a^This study was presented at ESC 2014 but has not been published.

AVB, atrioventricular block; AVN, atrioventricular node; BiVP, biventricular pacing; CRT, cardiac resynchronization therapy; HF, heart failure; HFH, heart failure hospitalization; LV, left ventricular; LVEF, left ventricular ejection fraction; LVEDD, left ventricular end-diastolic diameter; LVESV, left ventricular end-systolic volume; mo, months; NYHA, New York Heart Association functional class; QOL, quality of life; RCT, randomized controlled trial; RVP, right ventricular pacing; VP, ventricular pacing; yr, years.

#### Conduction system pacing as an alternative to biventricular pacing in patients with atrioventricular block

While HBP was first introduced in 2000,^31^ it was over the last decade that it gained popularity and widespread use due to the development of new tools that facilitated implantation. Left bundle branch area pacing was introduced somewhat later, with better results, higher success rates of implantation and better performance over time.^32^ Both techniques of CSP offer a theoretical advantage over BiVP of a more natural course of conduction of the paced activation into the left ventricle, and therefore better synchrony. While this is conceivable and may argue for CSP being offered to patients with need for long-term pacing in lieu of RV or BiVP, the evidence to support this claim remains scarce. Due to lack of sufficient data, the 2021 ESC guidelines^30^ did not recommend the use of CSP in patients in need of pacing with LVEF < 40% and did not make recommendations on LBBAP at all, as it was felt that the data were too limited, including data on long-term follow-up. According to the ESC guidelines, patients with heart failure and reduced ejection fraction are therefore recommended for biventricular CRT.^30^ The recent HRS consensus paper^14^ recommends physiological pacing (either BiVP or CSP) for a wider range of EF in cases of high burden RV pacing with a class of recommendation (COR) = 2a recommendation in patients with LVEF 36–50% and COR = 2b in patients with LVEF > 50%. In cases of AF who are destined for AV node ablation, BiV pacing has a COR = 2a (in patients with LVEF ≤ 50%) whereas CSP only has a COR = 2b (in any LVEF).

Despite rapidly growing research in the area since publication of the guidelines, up to now there are very few randomized controlled trials (RCTs) with reasonable duration of follow-up to support CSP as a substitute to BiVP in cases of AVB. They are listed in *Table 2* which also includes other studies on CSP for pacing indications. The majority are observational or case control studies with most of them are of modest size with <200 patients. Only three publications represent sizeable series. None of them is designed as a RCT with comparison of CSP to RV pacing or to BiVP in the population we are debating. The duration of follow-up is relatively short and the endpoints are soft in many of them.

**Table 2:** Main studies of CSP vs. RVP and CSP vs. BiV CRT pacing for AV block

Author/ref	Design	Inclusion criteria	*N* with CSP	Follow-up duration	Findings
Kronborg^33^	Cross-over comparison between RVP and HBP	Patients with AVB and LVEF > 40%	38	12 mo	Better LVEF, no clinical differences
Sharma^34^	Case control trial comparing to RVP	All paced patients with successful HBP	75	2 yr	Less HFH
Vijayaraman^35^	Comparison between RVP and HBP in two hospitals	All initially paced patients in two hospitals	75	5 y r	Better LVEF and lower HFH/mortality in a subgroup with > 40% pacing
Abdelrahman^36^	Comparison between RVP and HBP in two hospitals	All initially paced patients in two hospitals	304	754 d	Death, HFH, or upgrade less common in HBP group
Sharma^37^	Observational registry of LBBAP and RVP	Patients paced for bradycardia indications (65% AV conduction disease)	321	583 ± 274 d	Mortality + HFH + upgrade lower with LBBAP especially when VP > 20%
Tan^38^	2 center Observational registry of CSP vs. RVP	Consecutive patients implanted with pacemakers	231 (95 HBP, 136 LBBAP)	Median 432 days	HFH, upgrade to BiVP and all-cause mortality lower in CSP in patients with >20% pacing
Vinther^19^	RCT 1:1 HBP vs. BiVP	CHF, LVEF < 35% with LBBB	25	6 m	HBP similar to BiVP
His Alternative					
					High thresholds and crossovers in HBP group
Huang	Randomized cross-over between HBP and BiVP	Persistent AF with LVEF < 40 and narrow QRS undergoing AVN ablation	50	9 m	LVEF significantly better in HBP group
Alternative AF^39^					
Pujol-Lopez^18^	Case control trial CSP vs. BiVP	LVEF < 45% and AVB	27 (18 HBP, 7 LBBAP)	6 m	Similar response of EF, CRT response, better MR and NYHA improvement

AF, atrial fibrillation; AVB, atrioventricular block; AVN, atrioventricular node; BiVP, biventricular pacing; CRT, cardiac resynchronization therapy; CSP, conduction system pacing; d, days; HBP, His bundle pacing; HFH, heart failure hospitalization; LBBAP, left bundle branch area pacing; LBBB, left bundle branch block; LVEF, left ventricular ejection fraction; NYHA, New York Heart Association functional class; RCT, randomized controlled trial; RVP, right ventricular pacing.

Notably, there are already some published randomized trials that compare CSP to BiVP in patients with indications for CRT [usually HF and left bundle branch block (LBBB)], and they all point in the direction of CSP being better than BiVP in patients with LBBB and non-ischaemic cardiomyopathy.^18–20^ There are also several ongoing trials on this topic as summarized in a recent update.^40^

***Conclusion***—We therefore believe that evidence for CSP as pacing mode for AVB with narrow QRS and LVEF < 40% is not sufficient at this point to compete with the existing body of evidence supporting BiVP. The evidence supporting BiVP includes both larger numbers of randomized patients and longer follow-up.

## Contra

### Marek Jastrzebski, Kenneth Ellenbogen

*Paradigm shift in the treatment of atrioventricular block: from ameliorating symptoms and preventing sudden cardiac death, to restoring physiology*.

It is audacious to consider that the conduction system of the heart, developed over many millions of years of human evolution, might be functionally inferior to clinically established BiVP. Conduction system pacing ensures a synchronous ventricular depolarization pattern based on stimulation of the dense branching Purkinje fibre network that depolarize the ventricles rapidly at multiple endocardial sites, while BiVP utilizes typically two (more or less random) pacing points, with one of them being epicardial. Replacing a narrow QRS with non-physiological paced QRS, despite availability of a feasible and safe modality that can preserve the native conduction pattern, evidently goes against the Hippocratic oath of *primum non nocere*.

The universal goal of medical therapy and the standard approach to the management of any disease is to restore, as much as possible, the state of health and normal physiology that was present before the illness. Therefore, microsurgical re-implantation of the nerves and blood vessels of a severed limb, if feasible, is intuitively preferred to implanting a prosthetic limb. An analogous situation is present when the ventricular conduction system, responsible for synchronous activation of ventricular myocardium, is disconnected from the atria due to AVB. Intuitively, reconnection of the proximal conduction system should be the goal, especially in a patient with AVB and a narrow QRS. Implementation of a prosthesis to directly activate the working myocardium without engaging the conduction system, to partially mitigate the consequences of AVB, can only be justified if the above is not feasible, safe, or reliable. When pacing therapy was developed about 70 years ago, that was exactly the case. Medical technology was not advanced enough to offer anything more than single point right ventricular myocardial pacing. Although BiVP, developed approximately 25 years ago, was a major move forward with regard to the synchrony of ventricular contraction, it is still far from restoring normal physiology. The reasons for the continued use of RVP and BiVP in AVB patients, especially in those with a narrow QRS, must be seriously questioned today. The situation is nevertheless evolving, as CSP has been implanted in tens of thousands of patients worldwide.^41^

More physiological pacing modalities are comprised of right sided CSP which includes His bundle pacing (HBP) and right bundle branch pacing, and LBBAP which includes left fascicular pacing (LFP), left bundle branch pacing (LBBP), and left ventricular septal pacing (LVSP). Left ventricular septal pacing although, per definition, is not a CSP modality it is closely related, as indicated by similar paced QRS complex compared to LBBP QRS. This can be explained by prompt engagement of the left conduction system during LVSP with delay of only 10–20 ms, and, simultaneously, very slow initial spread of the direct depolarization of the working myocardium.

What is needed to adopt CSP as the preferred pacing modality in narrow QRS AVB patients with reduced LVEF, are mainly data on feasibility and safety, although studies indicating non-inferior or superior outcomes of CSP vs. BiVP are also important. Arguments for CSP will be reviewed below.

#### Biventricular pacing is suboptimal for atrioventricular block patients with narrow QRS

Recommendations for BiVP in patients with indications for ventricular pacing and heart failure are based on randomized studies that show that BiVP is better than RVP.^6^ An obvious reason for BiVP superiority is the known harmful impact of RV pacing in patients with AV block, as roughly 20% develop pacing-induced cardiomyopathy with RVP.^2^ Biventricular pacing in this population limits the harm of RVP rather than restores normal physiology. Biventricular pacing in narrow QRS patients always results in QRS prolongation that likely reflects introduction of some degree of electrical and therefore mechanical dyssynchrony. Importantly, there are no randomized studies that show that BiVP is better than maintaining a narrow QRS. On the contrary, there are several randomized trials (EchoCRT,^42^ LESSER-EARTH,^43^ and RethinQ^44^) and a meta-analysis,^45^ showing that BiVP is harmful when the native QRS is narrow. These studies on BiVP should be carefully interpreted in the current context because the enrolled patients did not have AVB. This is nevertheless a minor limitation, as native conduction was replaced by BiV pacing, as is the case in AVB. The only randomized study of CSP in AVB patients with reduced LVEF was HOPE-HF,^46^ which was conduction in patients with first-degree AVB. This was a randomized, cross-over, double-blind study which included patients with heart failure, LVEF ≤40%, PR ≥200 ms (average 249 ± 59 ms), and either QRS ≤140 ms or right bundle branch block (mean QRS 124 ± 26 ms) and compared HBP with no pacing. Although there was no difference in the primary endpoint of peak oxygen uptake, quality of life and patient preference were in favour of HBP. In another study randomized, cross-over, double-bling study comparing His/para-Hisian pacing with RV pacing in patients with QRS < 120 ms but LVEF > 0.40, LV function was significantly better with HBP.^33^

It is therefore reasonable to assume that maintaining a narrow QRS in AVB patients by delivering CSP is preferable to choosing BiVP. Nevertheless, randomized studies comparing these pacing modalities are needed.

#### Conduction system pacing in atrioventricular block patients is as feasible as biventricular pacing

The data on the feasibility of left CSP in AVB patients are derived from large and mid-sized multicentre observational studies. MELOS, the largest study to date, examined 2533 patients implanted with LBBAP at 14 European centres.^47^ In that study, 1218 patients with AVB were included. The reported success rate of LBBAP device implantation for bradyarrhythmia indications was 92.5% with an average fluoroscopy time of only 9 min. For patients with AVB, the success rates and fluoroscopy time were comparable at 91.1% and 8.5 min (unpublished data, available upon request). Moreover, this success rate included the learning curve of each centre. Similar LBBAP implantation success rate in 340/364 (93%) AVB patients was reported in a study from two US centres, without any difference between narrow and wide QRS patients.^48^ These success rates are comparable to those reported for BiVP, which for example it was 92.5% in MADIT-CRT.^49^ It seems that already with the limited spectrum of implantation tools, LBBAP is a faster procedure than BiVP and with less radiation exposure. Diaz *et al.*^50^ in a prospective multicentre study of 371 patients compared outcomes between LBBAP and BiVP as an initial implant strategy for CRT; fluoroscopy times were significantly shorter with LBBAP vs. BiVP: 95 min vs. 129, and 12 min vs. 21.7 min, respectively. Vijayaraman *et al.*^51^ reported the results from the International Collaborative LBBAP Study (I-CLAS) group—also showing shorter procedure duration, while fluoroscopy times were similar: 142 ± 55 min vs. 124 ± 48 min and 17 ± 15 min vs. 16 ± 12 min, for BiVP vs. LBBAP, respectively.

The average capture threshold during LBBAP is very similar to that seen with conventional RV pacing, for example—it was 0.6 V in MELOS^47^ and 0.5 V in the large dataset obtained from the Carelink™ (Medtronic, MN, USA) database.^41^ In MELOS,^47^ during follow-up of 6.4 ± 5.7 months the capture threshold remained stable with only 0.7%, 0.6%, and 0.16% of patients experiencing threshold rise over 1 V from baseline or to an absolute threshold value > 2.0 V or requiring lead repositioning due to threshold rise, respectively. In the Carelink™-based analysis 1.8% of patients had threshold rise to an absolute value > 2.0 V over 18 months of observation.^41^ This compares well to the capture thresholds during BiVP, and much more favourably to it when the total energy consumption is compared, because BiVP requires pacing from two ventricular sites while LBBAP is a single site pacing technique.

Sensing in the LBBAP position when programmed to bipolar is also comparable to RV pacing as the ring electrode ensures ample myocardium and is almost never an issue (average sensing of 10.6 mV in MELOS and 13 mV in the Carelink™ database).^41,47^

His bundle pacing is considered a more difficult technique compared with LBBAP. However, success rates for HBP in narrow QRS patients with AVB is similar to the success rate of LBBAP. Vijayaraman *et al.*^52^ reported implantation success rate of 93% and 76% of patients with nodal and infranodal AVB, respectively. HBP capture threshold (on average 1.6 ± 1.0 V/0.5 ms) was higher to that obtained with LBBAP, but still comparable to BiVP. HBP, although perceived as being more challenging for novice operators compared to LBBAP, is less invasive, results in much higher rate of confirmed conduction system capture, and offers perfect physiology with selective capture in narrow QRS patients. When dedicated implantation tools and/or more mature implantation technique for HBP are developed that eliminate late threshold rise and earlier battery depletion, it may become the preferred technique for narrow QRS AVB patients.

#### Conduction system pacing in atrioventricular block patients is as safe as biventricular pacing

Transseptal ventricular route of the pacing lead in LBBAP results in a different spectrum of complications compared to implanting the lead in the cardiac venous system for BiVP. However, the overall complication rate observed with LBBAP is comparable with the complication rate reported for BiVP, or lower. In the largest study comparing BiVP and LBBAP implanted for CRT indications (*n* = 1778), there were two times fewer complications in the LBBAP group (3.8% vs. 7.5%), mainly due to fewer infections and fewer lead dislodgements.^53^ In MELOS,^47^ the lead-related complications rates for LBBAP (8.3%) were comparable to the reported complication rate for BiVP.^6,54^

The dreaded acute complications of LBBAP are serious damage to the interventricular septum and late perforation into the LV cavity resulting in stroke. However, the rate of late perforation is extremely low, 0.08–0.33%, and there are no reports of lead-related strokes, despite hundreds of thousands of implanted leads.^47,55,56^ Rate of significant septal damage is also low, this manifests itself mainly as minor acute coronary event (0.4%) or septal haematoma (case reports only).^47,57^

With the current follow-up of 5 years since the first LBBAP implantation, the knowledge regarding the long-term performance of deep septal leads is limited. Extractability of LBBAP leads and long-term proarrhythmic effect of septal damage are potential concerns. However, pacing from cardiac veins is also associated with risks of extraction and potential ventricular proarrhythmia due to epicardial pacing.

In summary, although our knowledge on CSP complication profile is incomplete, especially with respect to long-term complications, currently it appears to be comparable to BiVP in frequency and clinical significance.

#### Conduction system pacing provides better electrical synchrony than biventricular pacing

The QRS complex is a recognized and straightforward biomarker of electrical synchrony. Average difference in QRS duration between BiVP and HBP is > 40 ms (125 ± 22 ms vs. 164 ± 25 ms in His-SYNC on-treatment analysis).^17^ That difference is slightly smaller between LBBP and BiVP because LBBP is characterized by a terminal R wave in V1 that prolongs QRS. In the LOT-CRT study, the difference in QRS duration between LBBP and BiVP was 26 ms: 144 ± 22 ms vs. 170 ± 30 ms, respectively.^58^ The largest study to date which compared LBBAP and BiVP QRS showed than LBBAP QRS is on average narrower by 16 ms (128 ± 19 ms vs. 144 ± 23 ms).^53^

**Figure 1:**
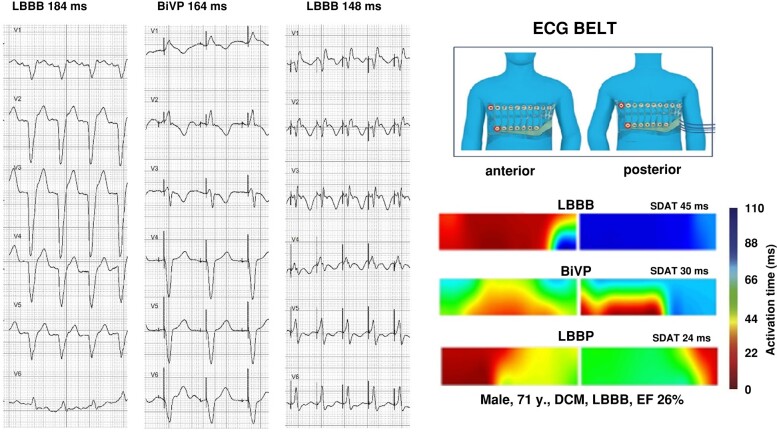
Data from 71-year-old patient with dilated cardiomyopathy (DCM) and left bundle branch block (LBBB). During biventricular pacing (BiVP), there is reduction of QRS duration from 184 to 164 ms and reduction in standard deviation of activation times (SDAT) obtained with the ECG Belt system from 45 to 30 ms. With left bundle branch pacing (LBBP), further reduction in QRS duration to 148 ms and of SDAT to 24 ms was observed.

Similar data, confirming superior electrical synchrony, were obtained with sophisticated mapping tools: ultra-high frequency ECG, high density body surface mapping and ECG belt technology. Ali *et al.* studied 14 patients with body surface mapping based on 252 surface electrodes (ECGi, Medtronic, Minneapolis, USA) and found an impressive difference between BiVP and CSP. In that study, HBP and LBBAP reduced left ventricular activation time (LVAT) much more than BVP (ΔLVAT for HBP –43 ± 16 ms, for LBBAP −45 ± 17 ms and for BiVP −13 ± 36 ms), with no difference between HBP and LBBAP.^59^ In the LEVEL-AT trial,^18^ a total of 66 patients were studied with ECGi; LBBAP and BiVP were found to result in a similar decrease of LVAT by 33 ms. Differences in these two studies might be related to different proportions of LVSP and LBBP/LFP in the LBBAP groups. Sussenbek *et al.*^60^ studied 80 patients with ultra-high frequency ECG, including assessment of e-DYS—a measure of dyssynchrony. LBBP led to significantly shorter e-DYS (24 ms) than BiVP (33 ms). Typical examples of 12-lead ECG and ECG belt results during LBBP vs. BiVP are presented on *Figure 1*.

Computational modelling and simulation showed that selective HBP best replicated physiological electrical activation and led to the most homogeneous mechanical behaviour, while LV septal pacing led to a slower and more heterogeneous LV activation than LBBP; BiVP led to synchronous LV–RV activation, but resulted in heterogeneous contraction.^61^

#### Conduction system pacing results in better haemodynamic performance of the heart than biventricular pacing

Early studies on haemodynamics of LBBAP reported that cardiac function in terms of LV stroke volume and LV dP/dT was better during single site LVSP than RV pacing and comparable to normal ventricular activation during sinus rhythm and BiVP.^62–64^ The increase in LVdP/dTmax was similar during LVSP and BiV pacing (17 ± 10% vs. 17 ± 9%, respectively) in one study.^64^ During LVSP the left conduction system is not captured directly but promptly engaged. This might result in inferior outcomes when compared to the confirmed capture of the left conduction system (LBBP/LFP) as indicated by some studies.^58,65^ Therefore, superiority of LBBAP over BiV might be expected mainly with confirmed capture of the left conduction system. Indeed, two recent studies comparing LBBP vs. BiV found greater haemodynamic improvement with LBBP.^59,66^ Liang *et al.*^66^ studied 21 patients with heart failure and LBBB; LBBP achieved a greater decrease in measures of dyssynchrony, and the increase in dP/dTmax from LBBP was significantly higher than that from BiVP. Ali *et al.*^59^ studied 12 patients and found greater improvement in acute invasive systolic blood pressure with HBP and LBBAP vs. BiVP: 8.1 ± 3.8 mmHg, 8.4 ± 8.2 mmHg and 6.4 ± 3.8 mmHg, respectively.

#### Data from randomized trials of conduction system pacing vs. biventricular pacing

There are no randomized studies comparing outcomes of CSP vs. BiVP in patients with narrow QRS and AVB. There are five small randomized studies of CSP vs. BiVP, but they all included predominantly LBBB patients—a different population, potentially less favourable for CSP than narrow QRS patients due to the potential for distal conduction system disease.^16–20^ With the limitations discussed above, these trials all showed non-inferiority or superiority of CSP compared with BiVP.

### Conclusion

On the basis of the available data, including large observational studies, CSP can be recognized as being a safe and feasible procedure with success rates and safety profile comparable to BiVP. Therefore, in patients with AVB and narrow QRS, on the basis of the principle of restoring or maintaining normal physiology and in line with the tenet of *primum non nocere*, using CSP to maintain physiologic activation of the ventricles should be preferred over BiVP, while awaiting supporting data from large randomized clinical trials. At the discretion of the operator and the consent of the patient, this pacing modality is already being used for AVB in many pioneering centres.

## Data Availability

There are no original data in this manuscript.
